# Importance of Analytical Thinking

**Published:** 1903-05

**Authors:** 


### Editorial.

### IMPORTANCE OF ANALYTICAL THINKING.

Reasoning powers have been given to every intelligent mind to weigh and analyze the subjects that are daily brought in contact with the mental powers. It is this that distinguishes man from the lower forms of animated life, and it is this which should dominate every action and serve the purpose of the creation,—that of being progressively in advance of all things below and combining therewith a continued aspiration for the ultimate—the perfected man.

To acquire this means an independent attitude to all subjects called for consideration, and not a cringing subservience to authority, however fortified that may have been in supposed facts. The history of mankind is simply a relation of experiences, and these aggregated make up our civilization as we understand it in this the twentieth century.

Dentistry has had its share in this onward march to a more enlightened goal, and we to-day are congratulating ourselves that we have made greater progress, in a limited time, than some of the callings that have made the world happier by their presence. This may or may not be true. That it has given a valuable service to humanity cannot be denied, and that it will continue to have a share in the upbuilding of world-blessings there can be no doubt, and for this we, as partners in this work, must be duly grateful. But are we all doing this in the best way?

This question will be answered, doubtless, by the assertion that the best way has not yet been discovered, and that, therefore, we must go on blindly seeking the true and direct path, trusting that in the decades yet to be we may perchance stumble upon it and march forward directly to the haven of perfection, if that beatific state can ever be reached in mortality.

The most important step in this direction seems to the writer to be in an independent state of mind or condition in which authoritative statements are to be considered of value in proportion as they coincide with reason and accepted scientific data.

Dentistry to-day needs thinkers as well as readers. The words are not synonymous. The individual may read as the voluble parrot talks, but an analysis of the concept of the author may be entirely wanting. The result is mental barrenness, and, however valuable the work may be, it falls, like the good seed, on unfruitful soil, and brings forth no fruitage for the good of the profession. On the other hand, the thoughtful, reasoning reader will find a mine of value in it and mentally add to his or her storehouse of information, laid aside for future use.

What use shall be made of this stored knowledge? This will depend on the individual. The man who bases his life on authority will find in it pabulum for future essays to be brought forth upon proper occasions. Another phase of mind will find, it may be, the mental force leading to deeper investigation, to a more earnest questioning of nature, to advance, if possible, one step nearer the substratum of truth.

This latter character of mind is most needed to-day in our profession. It is a gratification to feel that this class is gradually growing to be in the ascendant, and we have the result in more original productions than at any period in dentistry, and this is universal throughout the world, not being confined to any one locality or division of civilization.

There is, however, much to be desired still among the many in productions of more originality. The literature of our profession is still burdened by prolixity. Writers seem oftentimes to feel that quantity is most important in their productions, and that this takes precedence of quality. Padding, to use a common phrase, is resorted to until the readers weary with the commonplace, and the question is uppermost in the mind, Cannot writers find some subject, if not original, at least to treat it with the flavor of originality?

A marked evil in this direction is the tendency to quote extensively. There is but little attempt made by those who adopt this method to verify statements, or to attempt to produce similar results. The so-called facts are assumed, and then begins the building upon a weak foundation which will never support a superstructure. This dependence upon authority is a general human weakness, but the true scientist makes only a limited use of this for his foundation work. He who depends upon books or the thoughts of other minds leans upon a fractured staff. We need to draw our own water from the deep cisterns, and thereby appreciate the value of the labor and find out for ourselves whether the water is pure for the assuaging of our mental thirst.

The inherent tendency of most minds is to seek easy avenues of information. The undergraduate will use every effort to find some means to cram for examination. His elder brother, the practitioner, will do the same thing in another way, but both lazily hope to reach the end where superficial knowledge may serve instead of the analytical reasoning followed by an absorption of knowledge. In the long run the easy pathways usually lead into a wild and tangled mass of intellectual brushwood. The true solution is hard, thorough work and careful reasoning upon results, and, with this accomplished, the individual develops in mental stature.

The analytical mind is in one sense the creative mind, giving form to vague and seemingly unreal mental images. It is the antithesis of inaction; it earnestly seeks truth, never denies, nor will it accept assertion as equal to recorded fact. It has no room for mere faith, but cherishes hope, and is ever looking forward to the revelation of new possibilities in the realms of creative force.

This is education, whether it be in our calling or any other. Each has a special work to do in the world, and if this be done with the ever-mastering thought present that it must be done thoroughly, there will be no time for rest, neither will there be time to hunt up the dead past and depend upon it as the principal support, but rather to let it be as the dim rush-light of semi-illumined history, serving to render brighter, by contrast, the still darkened territory of future investigations. For this we must all work while the day is ours, and steadily advance towards the higher professional life.

